# Evaluating Google, Twitter, and Wikipedia as Tools for Influenza Surveillance Using Bayesian Change Point Analysis: A Comparative Analysis

**DOI:** 10.2196/publichealth.5901

**Published:** 2016-10-20

**Authors:** J Danielle Sharpe, Richard S Hopkins, Robert L Cook, Catherine W Striley

**Affiliations:** ^1^ College of Public Health and Health Professions Department of Epidemiology University of Florida Gainesville, FL United States; ^2^ Rollins School of Public Health Department of Epidemiology Emory University Atlanta, GA United States

**Keywords:** Internet, social media, Bayes theorem, public health surveillance, influenza, human

## Abstract

**Background:**

Traditional influenza surveillance relies on influenza-like illness (ILI) syndrome that is reported by health care providers. It primarily captures individuals who seek medical care and misses those who do not. Recently, Web-based data sources have been studied for application to public health surveillance, as there is a growing number of people who search, post, and tweet about their illnesses before seeking medical care. Existing research has shown some promise of using data from Google, Twitter, and Wikipedia to complement traditional surveillance for ILI. However, past studies have evaluated these Web-based sources individually or dually without comparing all 3 of them, and it would be beneficial to know which of the Web-based sources performs best in order to be considered to complement traditional methods.

**Objective:**

The objective of this study is to comparatively analyze Google, Twitter, and Wikipedia by examining which best corresponds with Centers for Disease Control and Prevention (CDC) ILI data. It was hypothesized that Wikipedia will best correspond with CDC ILI data as previous research found it to be least influenced by high media coverage in comparison with Google and Twitter.

**Methods:**

Publicly available, deidentified data were collected from the CDC, Google Flu Trends, HealthTweets, and Wikipedia for the 2012-2015 influenza seasons. Bayesian change point analysis was used to detect seasonal changes, or change points, in each of the data sources. Change points in Google, Twitter, and Wikipedia that occurred during the exact week, 1 preceding week, or 1 week after the CDC’s change points were compared with the CDC data as the gold standard. All analyses were conducted using the R package “bcp” version 4.0.0 in RStudio version 0.99.484 (RStudio Inc). In addition, sensitivity and positive predictive values (PPV) were calculated for Google, Twitter, and Wikipedia.

**Results:**

During the 2012-2015 influenza seasons, a high sensitivity of 92% was found for Google, whereas the PPV for Google was 85%. A low sensitivity of 50% was calculated for Twitter; a low PPV of 43% was found for Twitter also. Wikipedia had the lowest sensitivity of 33% and lowest PPV of 40%.

**Conclusions:**

Of the 3 Web-based sources, Google had the best combination of sensitivity and PPV in detecting Bayesian change points in influenza-related data streams. Findings demonstrated that change points in Google, Twitter, and Wikipedia data occasionally aligned well with change points captured in CDC ILI data, yet these sources did not detect all changes in CDC data and should be further studied and developed.

## Introduction

### Background

Although largely vaccine-preventable, influenza places a burden on the US health care system, causing 3000-50,000 deaths annually [1,2]. As one of the many influenza surveillance systems, the Centers for Disease Control and Prevention (CDC) monitors influenza activity by calculating the number of outpatient visits for the syndrome of influenza-like illness (ILI) reported by partnering health care providers to the US Outpatient ILI Surveillance Network (ILINet). The CDC defines ILI as a fever (≥100°F or 37.8°C) and a cough and sore throat without a known cause other than influenza [3]. This approach to surveillance primarily captures information about people who seek medical care for their influenza symptoms, thus missing those who do not interact with the health care system. In addition, this surveillance method is limited by relatively dated technology and by delays of up to 1 to 2 weeks between the occurrence of the illness event and the dissemination of surveillance information [4].

Syndromic surveillance, which can be defined as the monitoring of disease syndromes in or near real time for early detection of outbreaks, has incorporated the use of novel data sources such as emergency department records and prescription sales to enhance traditional surveillance systems [5-7]. Recently, nontraditional data sources, particularly those that are Web-based, have come into greater application for public health surveillance. This is especially evident as individuals who experience various symptoms may search the Web for health-related information and share their illness experiences using social media platforms before seeking medical care. Using such Web-based data sources such as search queries and social media has been coined digital epidemiology [8-10]. Digital epidemiology can be less expensive, timelier, and can expand detection by increasing the range of health events that can be detected.

### Related Work

As the number of Internet users has increased [11], researchers have identified the use of Google, Twitter, and Wikipedia as novel surveillance approaches to complement traditional methods. Google Flu Trends, which monitors Google users’ searches for information related to influenza, has shown correlation with CDC influenza data, while delivering estimates 1 to 2 weeks ahead of CDC reports [8,12]. Although initially successful, the system has not been without its issues in more recent years. Google Flu Trends overestimated influenza activity during the 2012-2013 influenza season and underestimated it during the 2009 H1N1 influenza pandemic [13-16]. One study found that both the original (2008) and revised (2009) algorithms for Google Flu Trends were not reliable on city, regional, and national scales, particularly in instances of varying intensity in influenza seasons and media coverage [16]. Due to issues with its proprietary algorithm, Google Flu Trends was discontinued in August 2015 [17].

Influenza-related posts on Twitter, a social networking platform for disseminating short messages (tweets), have shown high correlation with reported ILI activity in ILINet [18,19]. Studies have found that Twitter data highly correlate with national- and city-level ILI counts [20]. Signorini et al (2011) also demonstrated that tweets could be used to estimate ILI activity at regional and national levels within a reasonable margin of error [21]. Moreover, studies have found that Twitter data perform better than Google data. Nagar et al (2014) conducted a study showing that tweets better reflected city-level ILI incidence in comparison with Google search queries [22]. Aramaki et al discovered that a Twitter-based model outperformed a Google-based model during periods of normal news coverage, although the Twitter model performed less optimally during the periods of excessive media coverage [23]. Moreover, geographic granularity can affect the performance of Twitter data. Broniatowski et al (2015) found that city-level Twitter data performed better than state- and national-level Twitter data, although Google Flu Trends data performed better at each level [24].

Wikipedia page view data have proven valuable for tracking trending topics as well as disease monitoring and forecasting [25,26]. McIver and Brownstein (2014) reported that increases in the quantity of visits to influenza-related Wikipedia articles allowed for the estimation of influenza activity up to 2 weeks before ILINet, outperforming Google Flu Trends estimates during abnormal influenza seasons and periods of high media reporting [27]. One study found that Wikipedia page view data have suitable forecasting value up until the peak of the influenza seasons [26], whereas another study also reported that Wikipedia page view data are suitable for forecasting using a 28-day analysis as well as for nowcasting, or monitoring current disease incidence [25]. However, as a disadvantage, the signal-to-noise ratio of Wikipedia data can be problematic [25] as Wikipedia has become a preferred source for seeking health information whether an individual is ill or not [28,29]. In addition, unlike the granularity flexibility of Google and Twitter data, Wikipedia does not have such capability of evaluating influenza activity at local or regional levels because it only provides counts of page views and no accompanying location or user information in its publicly available data.

### Objective

These early studies on Google, Twitter, and Wikipedia show that, in spite of some drawbacks, mining these Web-based sources may provide valuable epidemic intelligence by identifying indicators of influenza activity at times or in populations that are missed by more traditional surveillance systems. Previous studies have evaluated these 3 Web-based sources individually or dually against a standard, but have not compared all 3 of them with each other and a standard. This comparison is needed to understand if each of these Web-based sources accurately reflect seasonal changes, or change points, that occur in CDC ILI data. It would be beneficial to know which of these Web-based sources performs the best in order to be considered as a complement to traditional surveillance methods.

Thus, this study aims to conduct a comparative analysis of using Google, Twitter, and Wikipedia for influenza surveillance by examining which Web-based source produces data that are most aligned with CDC ILI data. The specific research question is as follows: For which Web-based source—Google, Twitter, or Wikipedia—do detected change points most closely match change points detected in CDC ILI data for the 2012-2013, 2013-2014, and 2014-2015 influenza seasons? It is hypothesized that Wikipedia data will have the most change points in common with CDC ILI data due to McIver and Brownstein’s [27] finding that Wikipedia data can be less influenced by media coverage in comparison with data from Google Flu Trends and Twitter [16,23].

## Methods

### Data Collection

#### Study Period

Data were retrospectively collected for the US-designated 2012-2013 influenza season (September 30, 2012 to May 18, 2013), 2013-2014 influenza season (September 29, 2013 to May 17, 2014), and 2014-2015 influenza season (September 28, 2014 to May 23, 2015) [30-32]. This study period, that is 2012-2015, was chosen due to data constraints. The Twitter data from HealthTweets.org contained tweets dating back to November 2011. As we sought to analyze complete influenza seasons, we could not include the 2011-2012 influenza season, and therefore, any preceding seasons. In addition, we could not include data after the 2014-2015 influenza season because Google ceased making their Google Flu Trends data publicly available in August 2015.

All data were presented as Morbidity and Mortality Weekly Report (MMWR) weeks. MMWR weeks start on Sunday and end on Saturday, ranging from 1 to 52 or 53 weeks [33]. Each of the influenza seasons included in this study begins in MMWR week 40 of a year and ends in week 20 of the following year.

#### CDC Data

Data from the CDC ILINet system were downloaded from FluView Interactive, which provides weekly influenza surveillance information on outpatient illness, hospitalizations, pediatric mortality, virologic surveillance, and geographic activity [34]. ILINet count data are aggregated by MMWR week. The ILINet system aggregates weekly information from participating health care providers on counts of patients seen for ILI by age group, total patients seen by age group, and corresponding year and week [34]. Counts of ILI patient visits to the United States were used for this study. Although most prior studies used weighted ILI rates, we elected to use ILI counts. We decided to use CDC ILI count data to maintain unit comparison because we could not use the Bayesian change point analysis to transpose or model the Web-based count data to a similar scale as the CDC weighted ILI rates.

#### Google Data

Deidentified, national-level count data of influenza-related Google searches made in the United States were downloaded from the Google Flu Trends website [17]. These data are the output of a CDC data-fitted regression model and are based on Google Flu Trends’ 2009 model (for the 2012-2013 influenza season), 2013 model (for the 2013-2014 influenza season), and 2014 model (for the 2014-2015 influenza model) [17]. Count data from Google Flu Trends were already aggregated by MMWR week.

#### Twitter Data

For data from Twitter, deidentified, national-level count data of influenza-related tweets in the United States were downloaded from HealthTweets.org, a Johns Hopkins University-based repository of influenza-related tweets dating back to November 2011 [35]. Using the Twitter application programming interface (API), the HealthTweets team collected influenza-related tweets from a keyword stream, which is 1% of public tweets [35]. After collection, Dredze et al [28] categorized the influenza-related tweets using automated annotators based on keywords, keyword combinations, and the classifier developed by Lamb and colleagues [36]. Data from HealthTweets were also already aggregated by MMWR week.

#### Wikipedia Data

Wikipedia has made its article view data available for downloading through Wikimedia Statistics [37]. Wikipedia article view data that are deidentified and aggregated were gathered for views on the “Influenza” article (English version). Count data from the English version of the “Influenza” article served as a proxy for U.S. national-level Wikipedia views. Wikipedia data are presented as the number of article views by the hour, including nonunique views [37]. As Wikipedia article view data on the “Influenza” article are presented by the hour in Wikimedia Statistics, the data were aggregated by MMWR week before analysis.

### Statistical Analysis

#### Bayesian Change Point Analysis

Bayesian change point analysis was the method used for this study. In essence, this technique detects inflections that signal a change within time series data, also known as change points. Bayesian change point analysis has been primarily used to detect when significant changes occur within datasets that have big data properties, such as volume, variety, and velocity [38]. For instance, Bayesian change point analysis has been used to estimate when changes occurred in interest rate data [39], chromosomal microarray data [39], and cancer-related gene expression data [40]. This method was used to detect changes in emergency department attendance and hospital admissions after a health system transformation in a post-earthquake area [41]. Bayesian change point analysis has also been used to detect changes in the dynamics of an aquatic ecosystem such as the introduction of a nonnative species [42]. Besides our study, this Bayesian technique has been used only once for influenza surveillance using ILI visits to emergency departments [43], which is unlike our analysis in that we used Web-based data.

**Figure 1:**
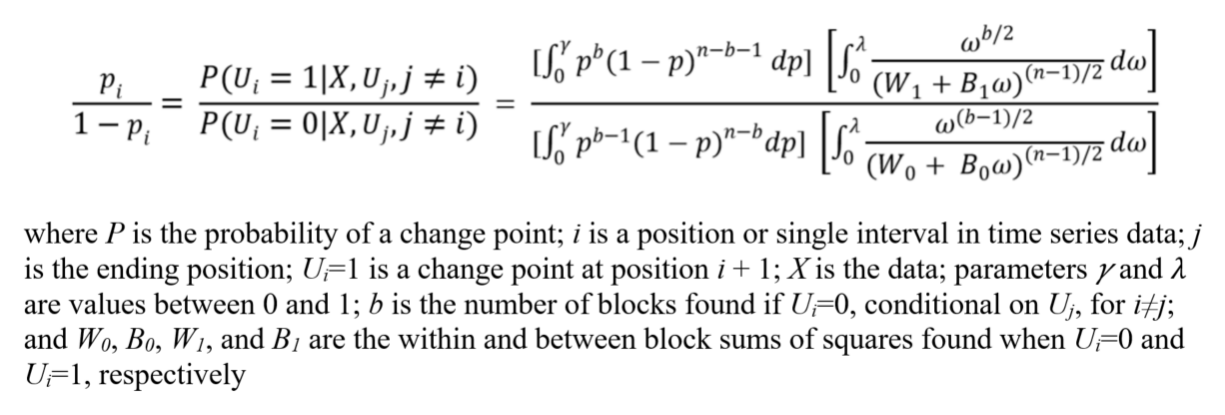
Simplified equation by Barry and Hartigan.

Bayesian change point analysis formed the method of choice as it is one of the proven methods that can detect subtle changes in time series data more effectively than traditional aberration detection methods [43]. Kass-Hout et al (2012) found that Bayesian change point analysis was not as sensitive as 2 other change point analysis methods—the cumulative sum technique and structural change model [43]. However, Bayesian change point analysis has been best applied to microarray data [39,40], which have big data properties similar to Web-based data. 

All Bayesian change point analyses were conducted using the R package “bcp” version 4.0.0 [39,40,44] in RStudio version 0.99.484 [45]. The “bcp” package implements a complex Markov Chain Monte Carlo (MCMC) approximation [39,40,44] of the Bayesian change point method described by Barry and Hartigan [46]. As the default for the “bcp” package, after 500 MCMC iterations, the probability of a change point at any given interval (ie, MMWR week) in time series data is computed from the number of times in the MCMC iterations that the condition of having a change point at that interval was met [39,40,44].

In each step of the Markov chain, the transition probability, *p*, for the conditional probability of a change point is found from the simplified equation by Barry and Hartigan [39,40,46], which is provided in Figure 1. After each MCMC iteration, the posterior means and probabilities are updated until the end of the time series. It is recommended that readers refer to Erdman and Emerson (2007), Erdman and Emerson (2008), and Barry and Hartigan (1993) for further mathematical explanation of this Bayesian method [39,40,46].

#### Change Points

We considered significant change points to be where the Bayesian method indicated the probability of a change occurring as ≥50%. Change points detected in the CDC ILI data were the gold standard with which change points found in the Web-based sources were compared. Change points of the Web-based sources that occurred during the exact week, 1 preceding week, or 1 week after the CDC change points were considered matching or true change points. This was done to account for any reporting lags that can be common with surveillance data. The number of change points for each data source was compared, and sensitivity and PPV for the detection of change points were calculated for each of the Web-based sources.

#### Sensitivity and Positive Predictive Values

Sensitivity and PPV were computed for each Web-based source using the change points detected for that Web-based source that matched change points detected for the CDC ILINet system (true positives), change points detected for the ILINet system but not for the Web-based source (false negatives), and change points detected for the Web-based source but not for the ILINet system (false positives). Sensitivity was calculated by dividing the true positives for each Web-based source by the total of true positives and false negatives, which would be the total number of CDC change points [47]. PPV were calculated by dividing the true positives for each Web-based source by the total of true positives and false positives, which would be the total change points for that particular Web-based source [47].

## Results

### Sample Characteristics

A summary of the count data that were collected and analyzed for the CDC, Google, Twitter, and Wikipedia for the 2012-2015 influenza seasons is provided (Table 1). There was year-to-year variability in the average weekly counts of events included for each of the data sources. For most of the data sources, the 2012-2013 influenza season had the highest average number of weekly counts. The 2013-2014 influenza season had the lowest average number for the CDC and Google Flu Trends, whereas the 2014-2015 influenza season had the lowest average number for Twitter and Wikipedia. Note that the 2014-2015 influenza season consisted of 34 total MMWR weeks because whereas most epidemiologic years are comprised of 52 MMWR weeks, the 2014-2015 epidemiologic year had 53 weeks due to a preceding calendar leap year. Table 1 further summarizes the data information.

**Table 1:** Summary of weekly Influenza-like Illness count data for the Centers for Disease Control and Prevention, Google, Twitter, and Wikipedia, 2012-2015 influenza seasons.

	Influenza season	CDC^a^ ILINet^b^	Google	Twitter	Wikipedia
**2012-2013**					
	MMWR^c^ Weeks (counts/week)	33	33	33	33
	Mean	19,049	4121	8096	47,541
	Min	7317	1286	2558	29,865
	Max	39,896	10,555	22,935	114,919
**2013-2014**					
	MMWR Weeks (counts/week)	33	33	33	33
	Mean	16,574	2274	5826	25,039
	Min	9033	1339	1196	17,885
	Max	28,654	5008	10,506	36,935
**2014-2015**					
	MMWR Weeks (counts/week)	34	34	34	34
	Mean	19,940	2549	2900	21,918
	Min	9289	1144	451	12,958
	Max	40,664	6911	8709	35,232

^a^CDC: Centers for Disease Control and Prevention.

^b^ILINet: United States Outpatient Influenza-like Illness Surveillance Network.

^c^MMWR: Morbidity and Mortality Weekly Report.

### Comparison of Change Points Detected in the 2012-2015 Influenza Seasons

**Figure 2:**
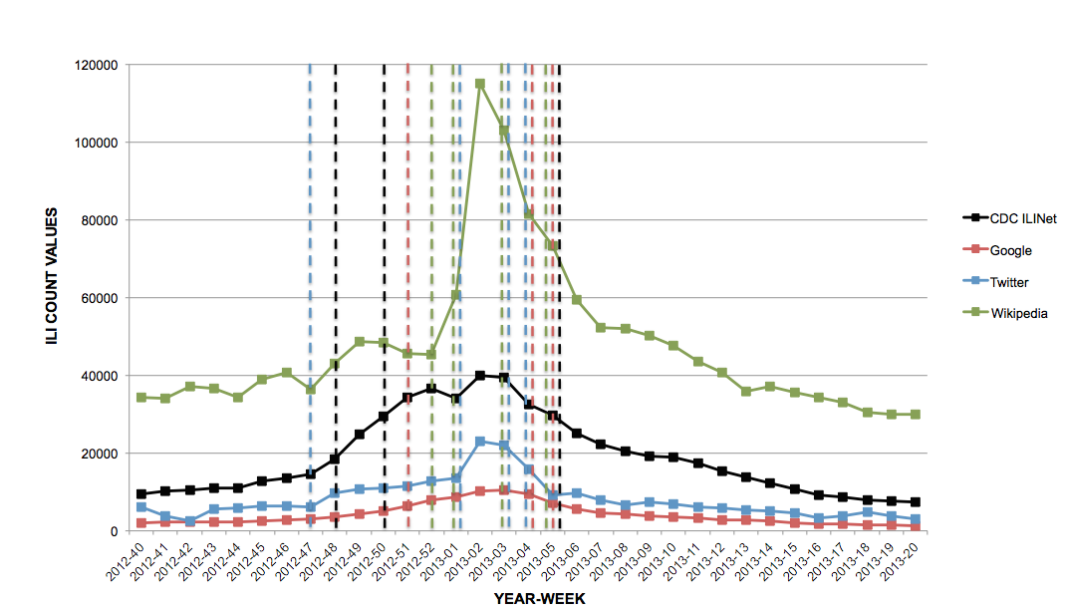
Change points (dotted lines) detected by Bayesian change point analysis, 2012-2013 influenza season.

**Figure 3:**
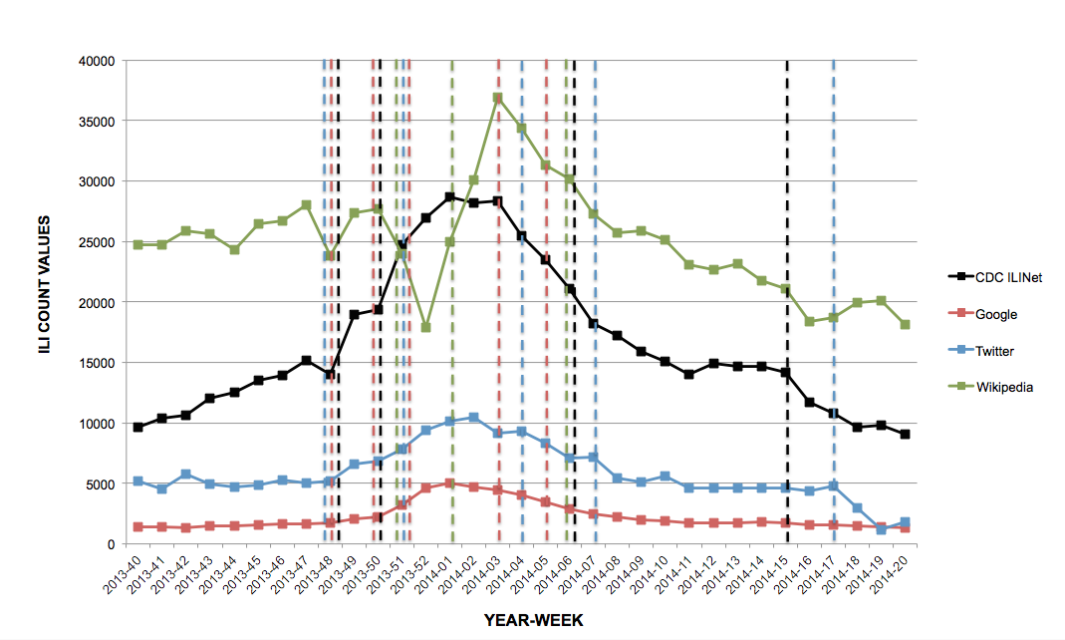
Change points (dotted lines) detected by Bayesian change point analysis, 2013-2014 influenza season.

**Figure 4:**
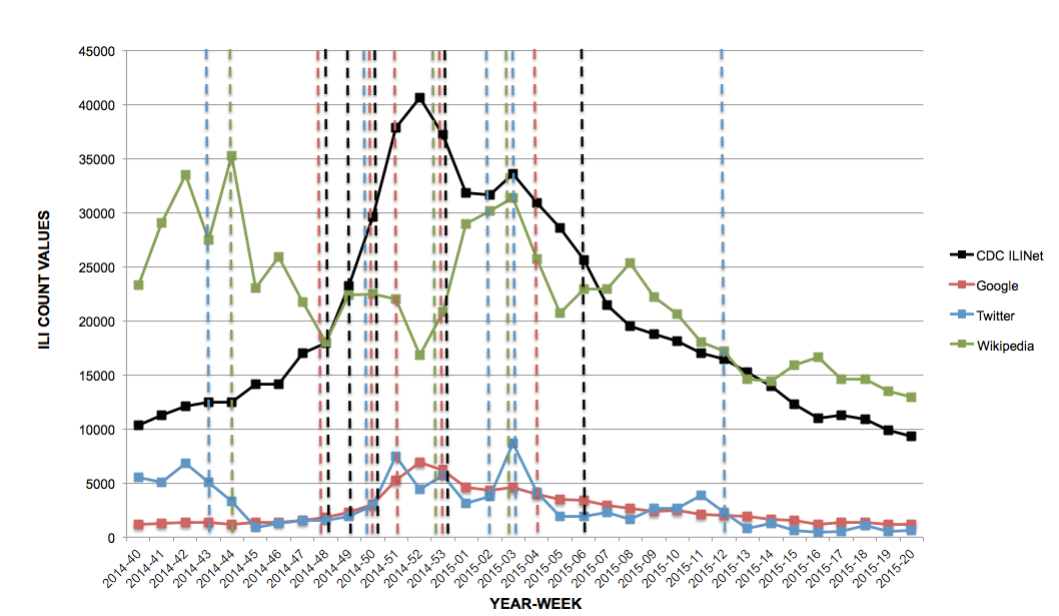
Change points (dotted lines) detected by Bayesian change point analysis, 2014-2015 influenza season.

A summary of all change points found in each data source is provided (see Figures 2-4), and a comparison of change points is shown in Table 2. For the 2012-2013 influenza season, Google had 3 total change points in common with the CDC ILINet system, which were MMWR weeks 51, 4, and 5. Twitter had 2 change points in common with the CDC’s change points, which were MMWR weeks 47 and 4. Wikipedia had only 1 change point that matched the CDC ILINet system, which was MMWR week 5.

In the 2013-2014 influenza season, Google had a total of 4 change points (MMWR weeks 48, 50, 51, and 5) that coincided with change points detected in the CDC data. Twitter had 3 change points (MMWR weeks 48, 51, and 7) that matched change points in the CDC ILINet system’s data. Wikipedia had 2 change points in common with CDC ILI data, which were MMWR weeks 51 and 6.

For the 2014-2015 influenza season, 4 change points (MMWR weeks 48, 50, 51, and 53) were detected in the Google data that concurred with change points identified in the CDC ILINet system. Both Twitter and Wikipedia had only 1 change point that coincided with the change points found in the CDC ILI data, which were MMWR weeks 50 and 53, respectively.

### Comparison of Sensitivity and Positive Predictive Value Detected Among Web-Based Sources

Next, we computed the sensitivity and PPV for each of the Web-based sources using the CDC ILI data as the gold standard. As shown in Table 3, results varied widely across the Web-based sources. A high sensitivity of 92% was found for Google, while the PPV for Google was 85%. A low sensitivity of 50% was calculated for Twitter; a low PPV of 43% was found for Twitter also. Wikipedia had the lowest sensitivity of 33% and lowest PPV of 40%. A table comparing sensitivity and PPV by specific influenza season is also provided (see Multimedia Appendix 1).

**Table 2:** Comparison of change points detected using Bayesian change point analysis, 2012-2015 influenza seasons^a^.

Influenza season	CDC^b^ ILINet^c^ counts (reference)	Google counts	Twitter counts	Wikipedia counts
**2012-2013**			Week 47^a^	
	Week 48			
	Week 50			
		Week 51^a^		
				Week 52
			Week 1	Week 1
			Week 3	Week 3
		Week 4^a^	Week 4^a^	
	Week 5	Week 5^a^		Week 5^a^
**2013-2014**	Week 48	Week 48^a^	Week 48^a^	
	Week 50	Week 50^a^		
		Week 51^a^	Week 51^a^	Week 51^a^
				Week 1
		Week 3		
			Week 4	
		Week 5^a^		
	Week 6			Week 6^a^
			Week 7^a^	
	Week 15			
			Week 17	
**2014-2015**			Week 43	
				Week 44
	Week 48	Week 48^a^		
	Week 49			
	Week 50	Week 50^a^	Week 50^a^	
		Week 51^a^		
	Week 53	Week 53^a^		Week 53^a^
			Week 2	
			Week 3	Week 3
		Week 4		
	Week 6			
			Week 12	

^a^MMWR week indicates a corresponding change point to the CDC change points (reference).

^b^CDC: Centers for Disease Control and Prevention.

^c^ILINet: United States Outpatient Influenza-like Illness Surveillance Network.

**Table 3:** Comparison of sensitivity and positive predictive value among Web-based sources, 2012-2015 influenza seasons.

Web-based source	Sensitivity (%)	Positive predictive value (%)
Google	92	85
Twitter	50	43
Wikipedia	33	40

## Discussion

### Principal Findings

Google had a total of 11 true change points (3 in the 2012-2013 influenza season, 4 in the 2013-2014 influenza season, and 4 in the 2014-2015 influenza season) that coincided with the CDC ILINet’s change points. As Google had the most change points that coincided with change points detected in the CDC ILI data, our hypothesis that Wikipedia would have the most change points was not supported. Sensitivity and PPV for event detection are important for evaluating the quality of surveillance systems [47]. Google had a moderate positive predictive value and was highly sensitive, whereas Twitter and Wikipedia both had low sensitivity rates and PPVs. This finding that Google had the best correspondence is not consistent with that of the previous studies that have found Twitter and Wikipedia to perform better [22,23,27,48].

Google, Twitter, and Wikipedia all had some change points that aligned well with CDC ILI data; however, they did not identify all change points that were identified in the CDC data, which would be important for understanding when seasonal changes occur during an influenza season. As no Web-based source identified all detected changes in the CDC data, this could indicate that the Web-based data, itself, may be limited in capturing all changes of CDC ILI data, which is quite plausible as not every individual who experiences ILI symptoms resorts to searching or sharing health information online. On the contrary, this could indicate that the Bayesian change point analysis as a technique is not adequately sensitive for the use on Web-based data. These 3 Web-based sources need to be further studied and compared using more standard statistical methods before being incorporated as surveillance data to complement a traditional system.

### Limitations

There are limitations of this study that should be noted. First, Bayesian change point analysis assumes time series data are distributed normally, which can be problematic as public health surveillance data can be variable and can have a nonnormal distribution [43]. However, we were unable to test this assumption on the “bcp” package in RStudio, and this is a limitation because the “bcp” package could have incorrectly identified or missed change points, especially if there were any outliers in the data to skew the Bayesian analysis. Another major limitation to using Bayesian change point analysis is that it cannot be used as a technique to monitor real-time data [49]. Bayesian change point analysis is best used to evaluate changes in historical time series data after all data have been collected. For this study, the Bayesian method was used to retrospectively evaluate data collected from the CDC, Google, Twitter, and Wikipedia after each influenza season occurred; therefore, the results cannot be directly applied for prospective use or real-time influenza surveillance.

A possible solution to conducting real-time influenza surveillance using Web-based data may lie in using a normal distribution algorithm. Normal distribution methods that are based on historical limits and cumulative sums have been traditionally used for influenza surveillance by the CDC [50]. Moreover, Pervaiz et al (2012) demonstrated that real-time influenza surveillance using Web-based data could be done more effectively using negative binomial- and Poisson-based models as opposed to normal distribution models due to the noisy nature of Web-based data and fluctuating numbers of Internet users and their activity levels [50].

Second, for the analysis of Wikipedia views, only the “Influenza” article was used for analysis, excluding other articles on influenza medications and influenza strains. McIver and Brownstein described the effectiveness of combining multiple influenza-related Wikipedia articles for surveillance purposes [27], but those were not included in this study. We assumed all views of the English-language Wikipedia “Influenza” article were by US users; however, some may have come from users in other English-speaking countries where the influenza season is very different, such as Australia.

Third, some of our data sources may have limits. We used CDC ILI count data for the analysis, which is not standard. Most prior studies have used weighted ILI rates instead of ILI counts because the weighted rates account for population variations in the United States. Using ILI counts may have sampling biases, but we justify the use of counts because we wanted to maintain data uniformity as none of the Web-based count data accounted for or could be normalized by population and regional variations in the United States. In addition, the Bayesian change point analysis did not allow us to transpose Web-based count data on the same scale as weighted ILI rates, thus ILI counts were the best option, considering the method used. Furthermore, the Google Flu Trends data used in this study were the output of a regression model that was fitted to CDC ILI data, leading to the Google data being a closer comparison with CDC ILI data. Although the Google Flu Trends data were fitted to match CDC data, it is important to note that these were readily available to the public as well as practitioners, justifying their use.

Fourth, data duplication could be an issue with each data source used in this study. Internet users can use a single website for multiple information searches and shares, and a single Internet user can use multiple websites for the same information search or share [51]. For example, a user can view the Wikipedia “Influenza” article multiple times and each view would be considered as a separate count [37]. Neither Google Flu Trends nor HealthTweets can distinguish or remove multiple searches and tweets by a single user [12,35]. In addition, there is no way, in publicly available data, to distinguish when a single user searches both Wikipedia and Google for the same information. The CDC ILINet system does not differentiate when a single patient makes repeated outpatient visits to the same participating health care provider or when a single patient makes outpatient visits to multiple health care providers for the same illness. This issue of data duplication should be further investigated in future studies.

Finally, Internet users are, on average, younger than the general U.S. population [52]. Although this difference may be viewed as a limitation to using Web-based data for influenza surveillance, younger age groups (0-4 years, 5-24 years, and 25-49 years) account for a majority of the outpatient ILI counts that are reported to the CDC ILINet system [34].

### Future Research

There is more substantive information in the content of Web-based sources that is not accounted for in count data of Web-based sources. Recent research has already begun to conduct content analyses of Web-based sources such as chat forums, Facebook, and Twitter in order to understand the health experiences and needs addressed by Internet users. Content analyses have proven valuable for both communicable and noncommunicable diseases because Internet users share and search about various health experiences ranging from mental health [53,54] to substance use [55,56] to the health needs of sexual minorities [57]. In addition, public health surveillance can be strengthened by combining various data sources, whether Web-based or traditional. Santillana et al (2015) found that when data from Google, Twitter, hospital records, and a participatory surveillance system were combined, influenza activity was predicted more accurately than and up to 4 weeks before the CDC [58]. More research should be carried out in this area to identify the best combination of traditional and novel data sources for influenza surveillance.

### Conclusions

To our knowledge, this is the first comparison to evaluate Google, Twitter, and Wikipedia as possible data sources for influenza surveillance against a common gold standard (the CDC ILINet system). Of the 3 Web-based sources, Google had the best combination of sensitivity and PPV in detecting Bayesian change points in influenza-related data streams. This finding is not consistent with existing research that has compared Google and Twitter data or Google and Wikipedia data, which could be attributed to the analysis of different influenza seasons, the novel use of the Bayesian method in this study, or the fact that Google Flu Trends data were fitted to CDC data. Further research should assess the substantive health content contained within these 3 Web-based sources, the surveillance value of combining these sources, and the ability of these sources to detect influenza activity using other statistical methods.

## Appendix

Multimedia Appendix 1 Comparison of sensitivity and positive predictive value (PPV) among web-based sources by specific influenza season.

## Abbreviations

API: application programming interface
CDC: Centers for Disease Control and Prevention
ILI: influenza-like illness
ILINet: United States Outpatient Influenza-like Illness Surveillance Network
MCMC: Markov Chain Monte Carlo
MMWR: Morbidity and Mortality Weekly Report
PPV: positive predictive value
