# Supplementary material for: Evaluating Google, Twitter, and Wikipedia as Tools for Influenza Surveillance Using Bayesian Change Point Analysis: A Comparative Analysis
Source: JMIR Public Health Surveill. 2016 Oct 20;2(2):e161. doi: 10.2196/publichealth.5901 (PMC5095368; doi:10.2196/publichealth.5901)
Supplement: Multimedia Appendix 1 [file publichealth_v2i2e161_app1.pdf]

Appendix 1

Table 4. Comparison of sensitivity and positive predictive value (PPV) among web-based sources by specific influenza season.

	2012-2013 influenza season		2013-2014 influenza season		2014-2015 influenza season	
	Sensitivity	PPV	Sensitivity	PPV	Sensitivity	PPV
Google	100%	100%	100%	80%	80%	80%
Twitter	67%	50%	75%	60%	20%	20%
Wikipedia	33%	25%	50%	67%	20%	33%
